# Phase field modelling of fracturing in frozen foods

**DOI:** 10.1016/j.crfs.2026.101437

**Published:** 2026-05-15

**Authors:** Ruud van der Sman

**Affiliations:** Wageningen-Food & Biobased Research, The Netherlands

**Keywords:** Fracture, Freezing damage, Simulation, French fries

## Abstract

In this study, we assess the fracture phase field method for simulating freezing damage in frozen foods. Inspired by Griffith’s theory of fracture, this method enables explicit modelling of the nucleation and growth of cracks in brittle materials. Par-fried French fries are chosen as a case study, for which we have previously demonstrated experimentally that freezing damage occurs. The fries are modelled as a composite material consisting of a starch matrix with embedded ice crystals. Fracture properties of the starch matrix as a function of moisture content and temperature are taken from an earlier study on cereal flakes; however, uncertainty remains regarding the fracture energy of ice crystals. At low values of the ice fracture energy, macroscopic cracks form at the crust–core interface, in contrast to experimental observations. At higher values, only microdamage is predicted. Simulations in which the freezing temperature and crust thickness are varied show good agreement with experimental trends. Overall, we consider the fracture phase field method to be a promising simulation tool for investigating fracture behaviour in food materials.

## Highlights

• First application of fracture phase field model to frozen foods.
• First model describing freezing damage in foods.
• Simulations results are qualitatively in line with experiments on dust formation.
• Fracture properties scale with Tg/T.

## Graphical abstract

**Figure:**
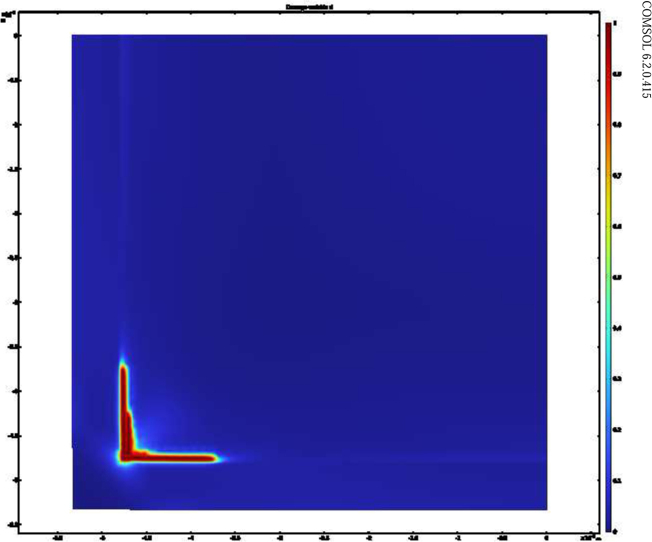

## 1 Introduction

Freezing significantly affects the quality of frozen foods (Van der Sman, 2020). Among the various quality aspects, freezing damage is one of the least understood phenomena (Dalvi-Isfahan et al., 2019). Only recently have new insights been gained due to advances in imaging techniques such as X-ray tomography and cryo-SEM. The physical causes of freezing damage are generally assumed to be: (i) volume expansion associated with the phase transition of liquid water to ice, (ii) strong variations in thermal and mechanical properties with ice fraction and temperature, and (iii) the development of stress gradients between the surface and core of the food during the different stages of freezing.

Freezing damage is even more critical in the fields of cryopreservation and cryosurgery, where it has therefore received substantially more attention (Rubinsky, 1992, Rabin et al., 1997, Han et al., 2009, Saeedi et al., 2025). For cryopreserved tissues, similar physical mechanisms are reported, often leading to crack formation (Saeedi et al., 2025). Freezing damage is also of major importance for plant survival (Pearce, 2001). In living tissues, viability requires that ice formation occurs extracellularly; however, this leads to cellular dehydration and damage to cell membranes, which constitutes another major mechanism of freezing injury (Yamada et al., 2002). For foods, cell membrane damage is mainly relevant for frozen fruits, as vegetables and animal tissues have typically lost membrane integrity prior to freezing due to blanching (Van der Sman, 2020) or post-mortem processes (Hamoen et al., 2013).

Freezing damage in foods is a multiphysics problem, in which heat and mass transfer and phase transitions are coupled to solid mechanics. We argue that a fundamental understanding of this phenomenon can only be achieved by developing multiphysics models alongside experimental research. At present, no models explicitly describing freezing damage in foods have been reported. Nevertheless, several studies have computed thermal stresses during freezing (Watanabe et al., 1996, Pham et al., 2005, Tremeac et al., 2007), which are then used as indicators for the probability of fracture. In addition, only a limited number of studies have focused on fracture of *unfrozen* food materials, with a few notable exceptions (Sridhar and Sommer, 2013, Skamniotis et al., 2017, Li et al., 2021, Samaras et al., 2023).

Modelling of the mechanics of frozen tissues is more advanced in the fields of cryopreservation and cryosurgery (Yang et al., 2008, Rabin, 2021, Solanki and Rabin, 2022, Krishna and Paul, 2023). Recently, these models have been extended with explicit descriptions of crack initiation and propagation using the fracture phase field method (Saeedi et al., 2025, Ying et al., 2026). The phase field approach is widely used in materials science (Miehe et al., 2010, Borden et al., 2012, Ambati et al., 2015, Wu et al., 2020), but it has not yet been applied to food materials, with the sole exception of Dammaß et al. (2024).

From a numerical perspective, the phase field method is particularly attractive compared to alternative approaches, as it does not require explicit crack tracking or remeshing during crack propagation, unlike the extended finite element method (XFEM), which has been applied to food materials in several studies (Sridhar and Sommer, 2013, Skamniotis et al., 2017, Li et al., 2021, Samaras et al., 2023). Moreover, crack nucleation naturally emerges from free-energy minimization. The method can also be readily combined with more advanced mechanical frameworks, such as large-deformation elastoviscoplasticity, and with multiphysics problems.

In this work, we therefore investigate the applicability of the fracture phase field method to modelling fracture in frozen foods caused by inhomogeneous volumetric expansion induced by ice formation. As a case study, we consider fracture in frozen par-fried French fries, which can lead to so-called dust formation during finish-frying (van der Sman, 2023, van der Sman and van den Oudenhoven, 2023). Similar physical mechanisms are involved in crust flaking during the baking of par-baked frozen dough (Le Bail et al., 2005, Dalvi-Isfahan et al., 2019). This phenomenon has previously been studied numerically (Fadhel Ben Aissa et al., 2008), but only thermal stresses were considered, without explicitly modelling freezing-induced damage. Coupling such models with the phase field approach enables this next step.

We build on existing phase field models developed for frozen systems, such as soils undergoing frost heave (Suh and Sun, 2022), concrete (Arasteh-Khoshbin et al., 2023), polar ice sheets (Sondershaus et al., 2024), and cryopreserved biological tissues (Saeedi et al., 2025, Liu et al., 2026). Fracture induced by solidification or freezing has been explicitly considered in Ying et al., 2026, Saeedi et al., 2025. In several of these applications, the fracture phase field method is coupled to viscoelastic large-deformation mechanics. In the present work, which represents a first application of the phase field fracture method to frozen food materials, we instead couple the model to small-deformation mechanics with brittle fracture, in order to limit the mathematical complexity of the multiphysics problem.

## 2 Model description

### 2.1 Fracture phase field method

We consider frozen food as a linear elastic material that can undergo brittle fracture. Within the phase field framework, the material behaviour is derived from a free-energy functional ψ, which consists of an elastic (mechanical) contribution $\psi_{m}$ and a fracture (damage) contribution $\psi_{d}$ (Carollo et al., 2018). The scalar damage phase field d represents the local damage state of the material, with 0≤d≤1, where d=1 corresponds to fully damaged material. The total free energy is given by

(1) $$\psi =g\left(d\right)\;\psi_{m}+\psi_{d}.$$

For a Hookean material with Lamé coefficients λ and μ, the elastic energy density reads

(2) $$\psi_{m}=\frac{1}{2}\lambda \;\text{tr}{\left(\epsilon \right)}^{2}+\mu \;\overset{¯}{\epsilon }:\overset{¯}{\epsilon },$$

where ϵ is the *elastic* strain tensor, and $\overset{¯}{\epsilon }=\epsilon -\frac{1}{3}\text{tr}\left(\epsilon \right)\;I$ denotes its deviatoric part.

The elastic energy is degraded by the function $g\left(d\right)={\left(1-d\right)}^{2}+\eta$, where η≪1 is a small numerical parameter ensuring a finite stiffness in fully damaged regions. This formulation implies that, as damage evolves, elastic energy is progressively reduced and converted into fracture energy.

The fracture energy is proportional to the crack surface area and represents the energetic cost of bond breaking. Within the phase field framework, this surface energy is described using a squared-gradient approximation (Borden et al., 2012, Carollo et al., 2018):

(3) $$\psi_{d}=\frac{G_{c}}{2L}d^{2}+\frac{G_{c}L}{2}{\left|\nabla d\right|}^{2}.$$

Here, L is a length-scale parameter related to the width of the diffuse crack, which must be smaller than any relevant physical dimension of the computational domain. The parameter $G_{c}$ is the specific fracture energy (J m^−2^), defined as the energy required to create a unit area of crack surface (Alvarez et al., 2000). It is directly related to the fracture energy appearing in Griffith’s theory of fracture.

The strong form of the phase field equation governing crack propagation then reads

(4) $$\frac{G_{c}}{L}\left(d-L^{2}\nabla^{2}d\right)=2\left(1-d\right)\;\psi_{m},$$

where the right-hand side represents the energetic driving force for damage evolution.

Since fracture occurs only under tensile loading, the elastic energy is decomposed into tensile and compressive contributions. Although (Miehe et al., 2010) proposed a spectral decomposition based on principal strains, this approach is often cumbersome to implement. Instead, a volumetric–deviatoric split is frequently adopted, as described in Amor et al. (2009). Accordingly, the elastic energy is written as

(5) $$\psi_{m}=\psi^{+}+\psi^{-},$$

with

$$\psi^{+}=\frac{\lambda }{2}{\langle \text{tr}\left(\epsilon \right)\rangle }_{+}^{2}+\mu \;\overset{¯}{\epsilon }:\overset{¯}{\epsilon },$$

(6) $$\psi^{-}=\frac{\lambda }{2}{\langle \text{tr}\left(\epsilon \right)\rangle }_{-}^{2}.$$

The Macaulay operators are defined as ${\langle x\rangle }_{+}=\max\left(x,0\right)$ and ${\langle x\rangle }_{-}=\min\left(x,0\right)$. In Eq. (4), the total elastic energy $\psi_{m}$ is replaced by its tensile part $\psi^{+}$.

Furthermore, in the standard phase field formulation it is assumed that fracture is irreversible and occurs only under tensile deformation. This irreversibility is enforced by replacing $\psi^{+}$ in the strong form with a history variable $H=\max_{t^{\prime }\leq t}\psi^{+}\left(t^{\prime }\right)$.

The split of the elastic energy also induces a corresponding split of the stress tensor:

(7) $$\sigma =g\left(d\right)\;\sigma^{+}+\sigma^{-},$$

with

$$\sigma^{+}=\lambda {\langle \text{tr}\left(\epsilon \right)\rangle }_{+}\;I+2\mu \;\overset{¯}{\epsilon },$$

(8) $$\sigma^{-}=\lambda {\langle \text{tr}\left(\epsilon \right)\rangle }_{-}\;I.$$

### 2.2 Heat transfer, ice formation, and thermal stresses

This section describes heat transfer, ice formation with its associated release of latent heat, and the resulting volumetric expansion leading to thermal stresses. In finite element modelling of solidification processes such as ice formation, it is convenient to represent the release of latent heat through a volumetric source term S (Celentano et al., 1994):

(9) $$\rho_{\mathrm{eff}}c_{p,\mathrm{eff}}\frac{dT}{dt}=\nabla \cdot \left(k\nabla T\right)+S.$$

Here, $\rho_{\mathrm{eff}}c_{p,\mathrm{eff}}$ denotes the effective volumetric heat capacity and k the thermal conductivity. Both quantities depend on the local composition, in particular the ice fraction, and are computed using the mixing rules described in van der Sman (2023).

The source term is derived from the enthalpy formulation (van der Sman, 2023):

(10) $$\frac{dH}{dt}=\nabla \cdot \left(k\nabla T\right),$$

where the enthalpy H is defined as

(11) $$H=\underset{i}{\sum }\phi_{i}\rho_{i}c_{p,i}\left(T-T_{\mathrm{ref}}\right)+\phi_{\mathrm{ice}}\Delta H_{\mathrm{ice}}.$$

Here, $\phi_{i}$ denotes the volume fraction of component i, with i=s,w,ice representing solids, unfrozen water, and ice, respectively. The quantity $\rho_{i}c_{p,i}$ is the volumetric heat capacity of component i, and $\Delta H_{\mathrm{ice}}$ is the latent heat of fusion of ice (in J m^−3^).

Applying the chain rule yields an explicit expression for the latent heat source term:

(12) $$\rho_{\mathrm{eff}}c_{p,\mathrm{eff}}\frac{dT}{dt}=\nabla \cdot \left(k\nabla T\right)-\Delta H_{\mathrm{ice}}\frac{d\phi_{\mathrm{ice}}}{dt}.$$

The evolution of the ice volume fraction is described using a simple relaxation model:

(13) $$\frac{d\phi_{\mathrm{ice}}}{dt}=\frac{\phi_{\mathrm{ice}}^{\mathrm{eq}}-\phi_{\mathrm{ice}}}{\tau },$$

where $\phi_{\mathrm{ice}}^{\mathrm{eq}}$ denotes the equilibrium ice fraction determined by the freezing curve.

We employ a simple relation between the initial freezing temperature $T_{m}$ and the amount of ice (van der Sman, 2023):

(14) $$\frac{y_{\mathrm{ice}}}{y_{w,0}}=\frac{T-T_{m}}{T-T_{f0}}\approx \frac{\phi_{\mathrm{ice}}}{\phi_{w,0}},\;\text{for}T<T_{m},$$

where $T_{f0}$ is the freezing point of pure water, $y_{i}$ denotes mass fractions, and $\phi_{w,0}$ is the volume fraction of water in the fully unfrozen state. The values of $T_{m}$ are taken to be the same as in our previous study (van der Sman, 2023).

The fries are treated as a composite consisting of unfrozen (supercooled) water, ice, and solids (starch and sucrose). We assume that starch and sucrose have identical density and thermal expansion properties. The evolution of the effective specific volume $v_{\mathrm{eff}}$ of the composite with temperature is computed assuming additivity of the specific volumes $v_{i}\left(T\right)=1/\rho_{i}$ of the individual components:

(15) $$\frac{1}{\rho_{\mathrm{eff}}}=v_{\mathrm{eff}}\left(T\right)=\phi_{\mathrm{ice}}\left(T\right)\;v_{\mathrm{ice}}\left(T\right)+\phi_{w}\left(T\right)\;v_{w}\left(T\right)+\phi_{s}\;v_{s}\left(T\right).$$

Temperature-dependent densities of the individual components are taken from Sippola and Taskinen, 2018, Gharsallaoui et al., 2008.

In the unfrozen reference state at $T_{\mathrm{ref}}=T_{f0}$, the volume fraction of water equals $\phi_{w,0}$. The volume fraction of unfrozen water evolves according to $\phi_{w}=\phi_{w,0}-\phi_{\mathrm{ice}}$. The specific volume in the reference state is denoted by $v_{\mathrm{ref}}=v_{\mathrm{eff}}\left(T_{\mathrm{ref}}\right)$. The inelastic thermal strain is related to volumetric expansion relative to the reference state as

(16) $$1+\epsilon_{\mathrm{th}}=J_{\mathrm{th}}^{\frac{1}{3}},\;J_{\mathrm{th}}=\frac{v_{\mathrm{eff}}\left(T\right)}{v_{\mathrm{ref}}}.$$

The total strains are derived from the displacement field u={u,v,w}. We consider a two-dimensional thermal stress problem in the cross section of a fry and therefore assume plane-stress conditions, such that $\sigma_{zz}=0$. The total strain components in the cross-sectional plane are given by

(17) $$\epsilon_{\mathrm{tot},ij}=\partial u_{i}/\partial x_{j}.$$

The elastic strain tensor is defined as

(18) $$\epsilon =\epsilon_{\mathrm{tot}}-\epsilon_{\mathrm{th}}\;I.$$

Under plane-stress conditions, $\sigma_{zz}=0$, which implies (Boley and Weiner, 2012)

(19) $$\epsilon_{zz}=\epsilon_{\mathrm{th}}-\frac{\nu }{1-\nu }\left(\epsilon_{xx}+\epsilon_{yy}-2\epsilon_{\mathrm{th}}\right),$$

where ν is the Poisson ratio, expressed in terms of the Lamé coefficients as

(20) $$\nu =\frac{\lambda }{2\left(\lambda +\mu \right)}.$$

For completeness, the relations between Young’s modulus E, Poisson’s ratio ν, and the Lamé coefficients are

$$\mu =\frac{E}{2\left(1+\nu \right)},$$

(21) $$\lambda =\frac{E\nu }{\left(1-2\nu \right)\left(1+\nu \right)}.$$

Note, the elastic strain tensor ϵ is the quantity entering the fracture phase field formulation.

### 2.3 Mechanical properties of frozen foods

Only a limited number of studies have modelled the mechanical properties of frozen foods as a function of ice fraction and temperature. To date, Shi et al. (1998) is one of the few studies that applied a simple parallel model to frozen potato tissue. Plant tissue can often be regarded as a hydrogel-like material (Jin and van der Sman, 2022), and the mechanical properties of frozen hydrogels have been investigated and modelled in detail in Yang et al. (2023). Although this latter work presents an improved model with good predictive accuracy, the formulation is mathematically rather complex. The authors also demonstrated that the Mori–Tanaka homogenization scheme provides reasonable predictions while remaining mathematically more tractable.

In these studies, ice crystals are assumed to grow preferentially in one direction, forming hexagonal columnar structures. For the cross section of a fry, the dominant growth direction of ice crystals is approximately normal to the surface. Given the nearly square cross section of a fry, we therefore assume that the effective mechanical properties can be treated as isotropic. Moreover, if french fries are properly frozen and stored at relatively constant temperature — the cellular tissue of potato keeps the ice crystal morphology rather isotropic, as shown by X-ray tomography (Zhao and Takhar, 2017).

Within the Mori–Tanaka homogenization scheme for isotropic frozen foods, the unfrozen tissue is treated as the matrix phase, while the ice crystals are modelled as elastic inclusions. Let $\phi_{\mathrm{ice}}$ denote the volume fraction of ice and $\phi_{m}=1-\phi_{\mathrm{ice}}$ the volume fraction of the matrix. The bulk and shear moduli of the matrix and inclusion phases are denoted by $\left(K_{m},G_{m}\right)$ and $\left(K_{i},G_{i}\right)$, respectively.

For an isotropic composite with randomly oriented inclusions, the effective bulk modulus $K_{\mathrm{eff}}$ is given by

(22) $$K_{\mathrm{eff}}=K_{m}+\frac{\phi_{i}\left(K_{i}-K_{m}\right)}{1+\left(1-\phi_{i}\right)\left(K_{i}-K_{m}\right)/\left(K_{m}+\frac{4}{3}G_{m}\right)}.$$

The effective shear modulus $G_{\mathrm{eff}}$ follows as

(23) $$G_{\mathrm{eff}}=G_{m}+\frac{\phi_{i}\left(G_{i}-G_{m}\right)}{1+\left(1-\phi_{i}\right)\left(G_{i}-G_{m}\right)/\left(G_{m}+f_{m}\right)},$$

where the auxiliary modulus $f_{m}$ is defined as

(24) $$f_{m}=\frac{G_{m}\left(9K_{m}+8G_{m}\right)}{6\left(K_{m}+2G_{m}\right)}.$$

The effective Young’s modulus $E_{\mathrm{eff}}$ and Poisson’s ratio $\nu_{\mathrm{eff}}$ are then obtained from the standard isotropic relations

(25) $$E_{\mathrm{eff}}=\frac{9K_{\mathrm{eff}}G_{\mathrm{eff}}}{3K_{\mathrm{eff}}+G_{\mathrm{eff}}},\;\nu_{\mathrm{eff}}=\frac{3K_{\mathrm{eff}}-2G_{\mathrm{eff}}}{2\left(3K_{\mathrm{eff}}+G_{\mathrm{eff}}\right)}.$$

The Young’s modulus of ice is assumed to evolve with temperature as (Yang et al., 2023)

(26) $$E_{\mathrm{ice}}\left(T\right)=E_{\mathrm{ref}}\left[1-\gamma \left(T-T_{\mathrm{ref}}\right)\right],$$

with γ=0.05 K^−1^. At $T_{\mathrm{ref}}=273$ K, the modulus equals $E_{\mathrm{ref}}=0.45\;\mathrm{GPa}$, and the Poisson ratio is $\nu_{\mathrm{ice}}=0.32$.

The Poisson ratio for frozen potatoes is reported as ν≈0.35, which is close to values reported for both pure ice ($\nu_{\mathrm{ice}}=0.32$) (Yang et al., 2023) and the unfrozen matrix ($\nu_{m}=0.32$) (Ross and Scanlon, 2004). We therefore assume a constant Poisson ratio over the full temperature range. This allows an adapted Mori–Tanaka procedure: (i) compute the effective shear modulus $G_{\mathrm{eff}}$ from the homogenization scheme, (ii) obtain the effective bulk modulus $K_{\mathrm{eff}}$ from $G_{\mathrm{eff}}$ and the assumed constant ν, and (iii) compute the Lamé coefficients.

For the unfrozen matrix phase, we assume that the mechanical properties are similar to those of cereal flakes. This material is also starch-rich, and literature data are available for both Young’s modulus E and the fracture energy $G_{c}$. From this dataset we determine the dependence on temperature and moisture content. In the spirit of our earlier studies (Van der Sman et al., 2022, van der Sman et al., 2026), we analyse whether the mechanical properties scale with $T_{g}/T$, where $T_{g}$ denotes the glass transition temperature.

For (sweetened) corn flakes, Georget and Smith (1996) investigated fracture properties, quantified by $G_{c}$, in relation to the glass transition temperature $T_{g}$ and Young’s modulus E. We test the hypothesis that both $G_{c}$ and E can be expressed as functions of $T_{g}/T$. The cereal samples were studied over a range of water contents and for different plasticizers (fructose and sucrose). Glass transition temperatures were reported and fitted using the Couchman–Karasz equation, exploiting that $\Delta c_{p,s}$ is similar for proteins, starch, and sugars (Van der Sman, 2016).

The fracture energy of ice inclusions is not available in the literature (Saeedi et al., 2025). For frozen beef, values in the range $100<G_{c}<200$ J m^−2^ have been reported (Dobraszczy et al., 1987), reflecting the combined contributions of the matrix and ice. For polycrystalline ice, a much lower value $G_{c}\approx 2$ J m^−2^ has been assumed (Suh and Sun, 2022). Ice inclusions in potato tissue are expected to be predominantly mono-crystalline and therefore stronger than polycrystalline ice. We therefore assume an intermediate value, $G_{c,\mathrm{ice}}=10$ J m^−2^.

Mixing rules for $G_{c}$ are not well established in the literature. We therefore assume a Hill average of the parallel and series models (Hill, 1952), corresponding to the upper and lower Voigt–Reuss bounds:

$$G_{c,\mathrm{eff}}=\frac{1}{2}\left(G_{c,∥}+G_{c,⊥}\right),$$

$$G_{c,⊥}=\phi_{\mathrm{ice}}\;G_{c,\mathrm{ice}}+\left(1-\phi_{\mathrm{ice}}\right)\;G_{c,m},$$

(27) $$\frac{1}{G_{c,∥}}=\frac{\phi_{\mathrm{ice}}}{G_{c,\mathrm{ice}}}+\frac{1-\phi_{\mathrm{ice}}}{G_{c,m}}.$$

### 2.4 Implementation

All models described above are implemented in COMSOL using the weak-form interface. We apply the coupled formulation to a two-dimensional cross section of a par-fried fry during freezing, assuming plane-stress conditions.

The weak form of the momentum balance for small deformations is based on the virtual work of the stress:

(28) $$\int_{\Omega }\sigma :\nabla \overset{\sim }{u}\;d\Omega ,$$

where $\overset{\sim }{u}$ denotes the test function. In the fracture phase field formulation, the momentum balance is degraded by multiplication with g(d). In the 2D COMSOL implementation this yields the contributions

(29) $$-\;g\left(d\right)\left[\sigma_{xx}\;\partial_{x}\overset{\sim }{u}+\sigma_{xy}\;\partial_{y}\overset{\sim }{u}\right]-\;g\left(d\right)\left[\sigma_{xy}\;\partial_{x}\overset{\sim }{v}+\sigma_{yy}\;\partial_{y}\overset{\sim }{v}\right].$$

Mind, that $\sigma_{ij}$ represents the total stress components, which are splitted following Eq. (7).

The weak form for the damage phase field follows from the stationarity of the total free energy and reads

(30) $$\int_{\Omega }\left(\frac{\partial g\left(d\right)}{\partial d}\;\psi_{0}\;\overset{\sim }{d}+\frac{G_{c}}{L}\;d\;\overset{\sim }{d}+G_{c}L\nabla d\cdot \nabla \overset{\sim }{d}\right)\;d\Omega ,$$

with $\overset{\sim }{d}$ the test function associated with the damage field. In the 2D COMSOL implementation we use

(31) $$\overset{\sim }{d}\;\overset{˙}{d}\;\eta_{d}-2\left(1-d\right)\;\overset{\sim }{d}\;H+\frac{G_{c}}{L}\;d\;\overset{\sim }{d}+G_{c}L\left(\partial_{x}d\;\partial_{x}\overset{\sim }{d}+\partial_{y}d\;\partial_{y}\overset{\sim }{d}\right).$$

A small viscous regularization term (linear in the time derivative $\overset{˙}{d}$) is added such that the phase field equation is solved in a quasi-steady manner. Here, $\eta_{d}$ is a numerical parameter; this additional term improves numerical stability.

Recall that H denotes the history field. For improved numerical stability, we solve an evolution equation for H:

(32) $$\partial_{t}H=\frac{\max\;\left(\psi^{+}-H,\;0\right)}{\tau_{H}}.$$

Note, in the limit of $\tau_{H}\to 0$ the relaxation equation goes towards the normal definition of the history field H. In the implementation $\tau_{H}$ is kept smaller than any relevant physical time scale.

The weak form for the energy balance reads:

(33) $$\int_{\Omega }\rho_{eff}c_{p,eff}\overset{˙}{T}\overset{\sim }{T}-k\nabla T\cdot \nabla \overset{\sim }{T}-S\overset{\sim }{T}d\Omega$$

Mind that $S=-\Delta H_{ice}{\overset{˙}{\phi }}_{ice}$. The ice volume fraction $\phi_{ice}$ is obtained via solving the ordinary differential equation, Eq. (13).

To resolve the diffuse crack, the mesh size must be smaller than L. The numerical parameters are chosen as $\eta =10^{-5}$, L=25μm, $\tau_{H}=1$ s, and $\eta_{d}=16000$.

For time integration and spatial discretization we used the default COMSOL settings. Spatial discretization is performed with quadratic Lagrange elements.

We simulate one quarter of the square cross section. Symmetry boundary conditions are applied at x=0 and y=0. At the outer boundary, natural boundary conditions (zero traction/zero flux) are applied for the momentum balance and the phase field. For the energy balance, Robin boundary conditions are prescribed:

(34) $$-k\nabla T\cdot n=h_{\mathrm{air}}\left(T-T_{\mathrm{air}}\right),$$

where n is the outward unit normal. The heat-transfer coefficient is set to $h_{\mathrm{air}}=140$ W m^−2^ K^−1^, representative of shock freezing conditions, and $T_{\mathrm{air}}$ denotes the freezer temperature.

**Fig. 1:**
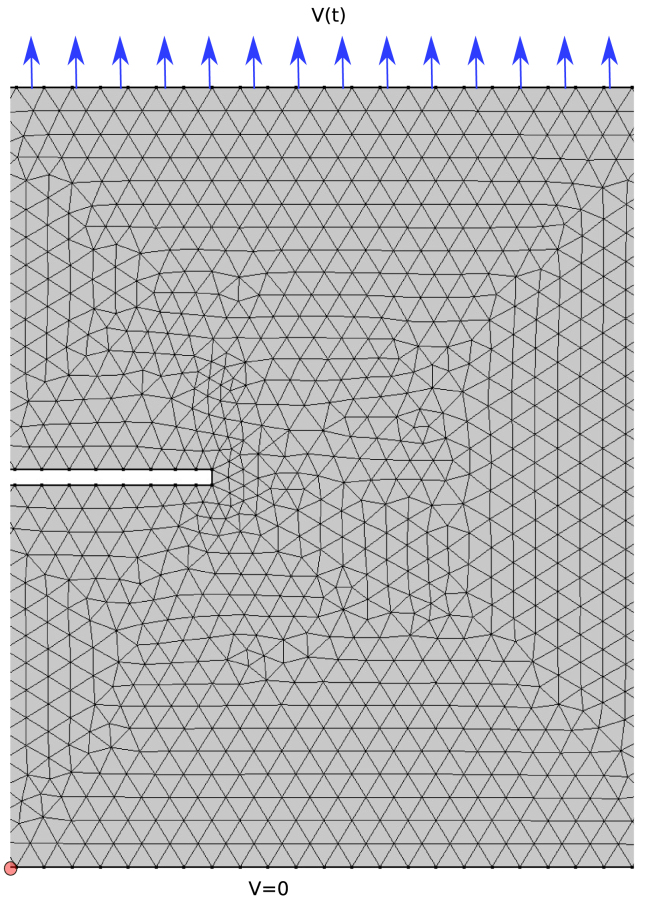
Geometry and mesh of the side-notch benchmark. The square domain contains a thin notch at the centre of the left boundary. The vertical displacement at the bottom boundary is constrained (v=0), and the lower-left corner is fixed (u=v=0). A prescribed vertical displacement $v_{\mathrm{load}}\left(t\right)$ is applied at the top boundary. Natural (traction-free) boundary conditions are applied on the remaining boundaries.

## 3 Results

### 3.1 Side-notch benchmark

We first verify the consistency of the weak formulations using a well-known benchmark problem in phase field fracture modelling: crack propagation in a side-edge-notch test. The benchmark consists of a square domain with a notch located at the centre of the left boundary. A vertical displacement $v_{\mathrm{load}}\left(t\right)∼t$ is prescribed at the top boundary, corresponding to a constant loading rate. At the bottom boundary, the vertical displacement is fixed to zero, v(y=0)=0. To eliminate rigid-body motion, one point at the bottom-left corner is fully constrained, u(x=0,y=0)=0. All remaining boundaries are subject to natural (traction-free) boundary conditions. The geometry and mesh are shown in Fig. 1.

For the damage phase field, zero-flux boundary conditions are imposed on all boundaries. The history field is initialized as H(x,y,t=0)=0, and the initial displacement field is zero. The notch is explicitly cut out of the square geometry. The damage field is initialized uniformly as d(x,y)=0, such that crack nucleation occurs spontaneously during loading.

**Fig. 2:**
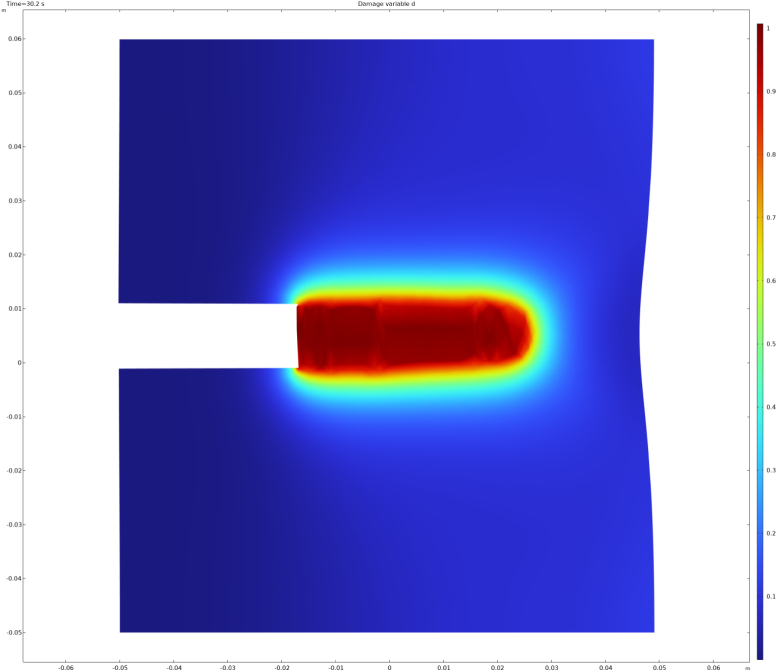
Simulation result showing a contour plot of the damage field d on the deformed domain. The fracture corresponds to d=1 and propagates horizontally from the notch towards the right boundary.

The simulation result after 30 s is shown in Fig. 2. Clear propagation of the fracture from the tip of the notch towards the right boundary of the domain is observed. The damage field is shown as a contour plot on the deformed configuration. The notch widens due to the imposed vertical displacement, while the right boundary bows inward as a result of material degradation caused by the advancing crack. This behaviour is consistent with previously reported simulations of this benchmark problem (Miehe et al., 2010). At later times, the simulation becomes unstable as the crack approaches the right boundary of the domain.

### 3.2 Fracture properties of glassy starch

**Fig. 3:**
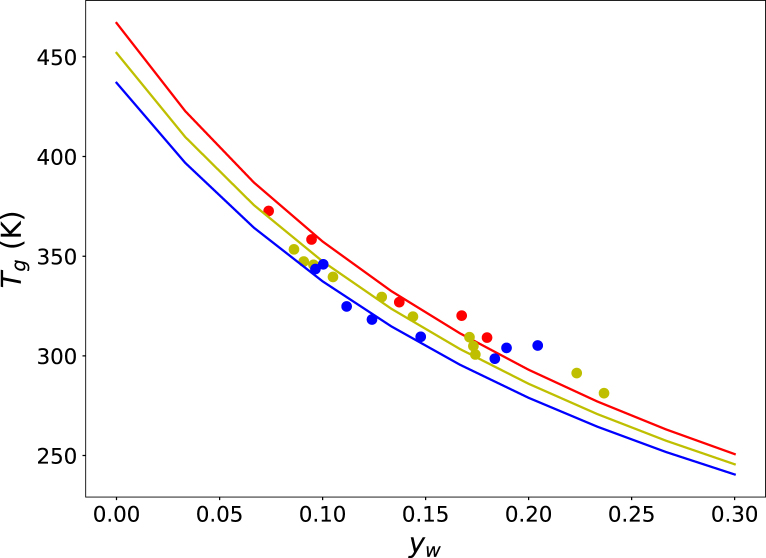
Glass transition temperatures of cereal flakes at different moisture contents (red) and with added fructose (yellow) or sucrose (blue). Experimental data are shown as symbols, while solid lines represent the Couchman–Karasz fits.

We tested the $T_{g}/T$ scaling of mechanical and fracture properties of starch-rich materials using the experimental data reported in Georget and Smith (1996). First, the glass transition temperatures were fitted using the Couchman–Karasz equation for three different plasticizer formulations, as shown in Fig. 3. The resulting fitted relations were then used to compute the corresponding values of $T_{g}/T$ under the conditions of the mechanical tests.

**Fig. 4:**
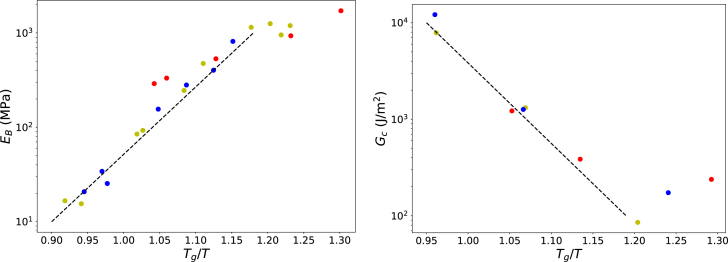
Young’s modulus E (left panel) and fracture energy $G_{c}$ (right panel) of cereal flakes as functions of $T_{g}/T$. Dashed lines are guides to the eye.

Fig. 4 shows that, when plotted as a function of $T_{g}/T$, the data collapse onto a master curve, in contrast to the original representation as a function of moisture content alone. For $T_{g}/T>1.2$, both the Young’s modulus E and the fracture energy $G_{c}$ appear to approach constant plateau values. For $T_{g}/T<1.2$, log(E) and $\log\left(G_{c}\right)$ vary approximately linearly with $T_{g}/T$, albeit with opposite slopes. Moreover, the product $E\;G_{c}$ appears to be approximately constant over the full range of conditions considered.

This collapse of data is consistent with the findings of Gent and Lai (1994), who demonstrated that fracture energy exhibits time–temperature superposition when using Williams–Landel–Ferry (WLF) scaling, commonly applied to viscosity. For carbohydrates and polysaccharides, however, we have previously shown that $T_{g}/T$ scaling remains valid over a much wider range than WLF scaling when applied to viscosity data (Van der Sman and Mauer, 2019, van der Sman et al., 2026, Van der Sman et al., 2022).

### 3.3 Fracturing of frozen par-fried potato strips

We investigate freezing-induced damage in par-fried potato strips, focusing on fries with a square cross section of 10 mm × 10 mm. Par-frying results in dehydration of the crust. In the simulations, the crust is assumed to have a constant thickness of 0.5 mm, while its moisture content varies as a consequence of the par-frying process. Freezing is assumed to start immediately after par-frying. The freezing process is characterized by the processing time $t_{e}$ and the external freezer temperature $T_{\mathrm{ext}}$.

**Fig. 5:**
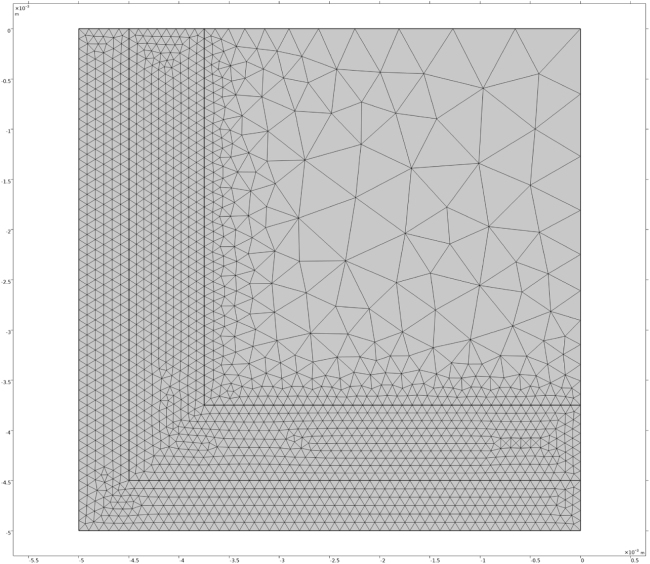
Mesh used for calculations of freezing damage to fries.

**Fig. 6:**
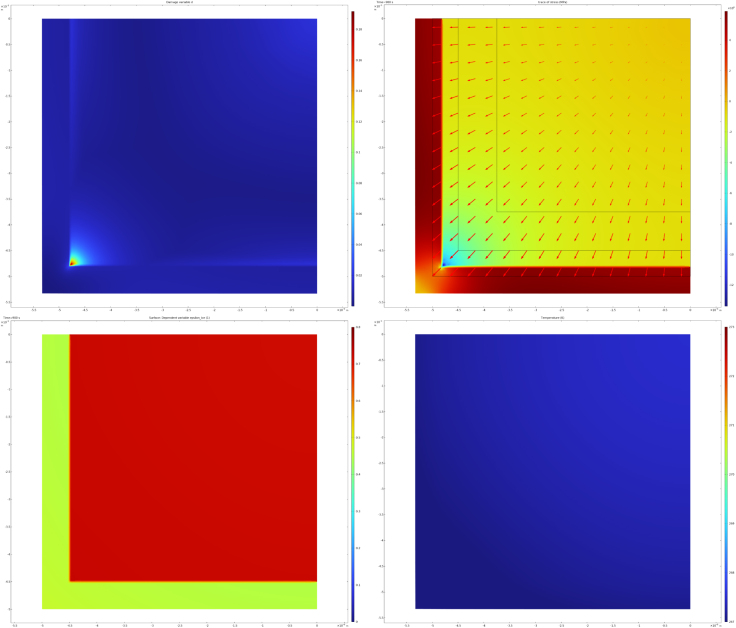
Computed state variables at the end of freezing ($t=t_{e}$): (a) damage field d, (b) (von Mises) stress field, (c) ice volume fraction, and (d) temperature field.

A first set of simulations is performed for $t_{e}=900$ s and $T_{\mathrm{ext}}=-8{\;}^{\circ }C$. The meshing used for these simulations is shown in Fig. 5. The moisture content of the core is set to $y_{w}=0.775$, while that of the crust is $y_{w}=0.64$. The initial freezing temperatures, which depend on composition (sugar and starch content) and moisture content, are taken as $T_{m}=271.5$ K for the crust and $T_{m}=272.8$ K for the core. These values are used as input for the ice fraction–temperature relation in Eq. (14). To illustrate the simulation results, contour plots of several state variables are shown at selected times. The distributions of all state variables at the end of the freezing process are presented in Fig. 6. In the stress plots, the deformation field is superimposed using displacement vectors.

**Fig. 7:**
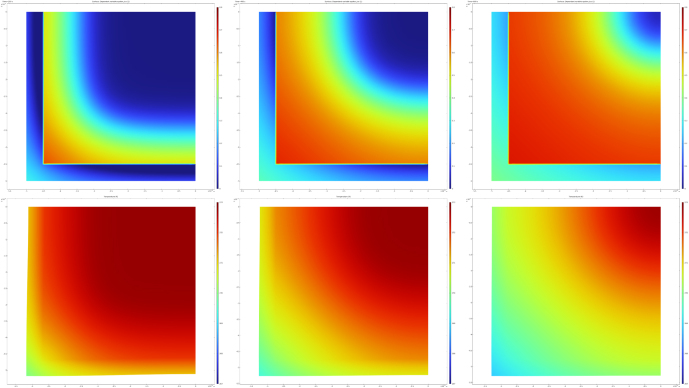
Time evolution of ice fraction (top) and temperature field (bottom) at t=200, 400, 600 s (from left to right).

The temporal evolution of the ice fraction and temperature fields is shown in Fig. 7. Due to its lower initial moisture content, less ice forms in the crust than in the core. As a result, the core undergoes a larger volumetric expansion, placing the partially frozen crust under tensile stress, while the core itself is predominantly under compression. Localized damage develops at the interface between crust and core, with particularly high concentrations near the sharp corners of the crust–core interface. However, the damage variable does not reach its critical value, indicating that no macroscopic fracture occurs during freezing. The actual fracturing happens during finish-frying, and this pre-dominantly happens at the crust/core interface (Gouyo et al., 2021) - which has been weakened by the microdamage induced by the freezing.

This observation is consistent with our earlier hypothesis that freezing primarily induces microdamage, which only develops into macroscopic fractures during subsequent finish frying. After 900 s of freezing, a steady state in both temperature and ice fraction is reached, as shown in Fig. 7. Ice formation initiates earlier in the core and occurs later in the crust due to the difference in initial freezing temperatures. This difference is also evident in the temperature fields at t=200 s and t=400 s.

Next, we examined the sensitivity of the predicted freezing damage to numerical and physical parameters. The numerical solution is not very sensitive to the rate-controlling parameters $\eta_{d}$ and $\tau_{H}$, which can be varied safely within the ranges $200\leq \eta_{d}\leq 16000$ and $1\leq \tau_{H}\leq 10$ s. In contrast, the solution depends on the regularization length scale L. The literature suggests choosing $L\approx \Delta x_{\min}/4$, where $\Delta x_{\min}$ denotes the smallest grid spacing. A proper convergence study therefore requires simultaneous refinement of both L and the spatial discretization Δx.

**Fig. 8:**
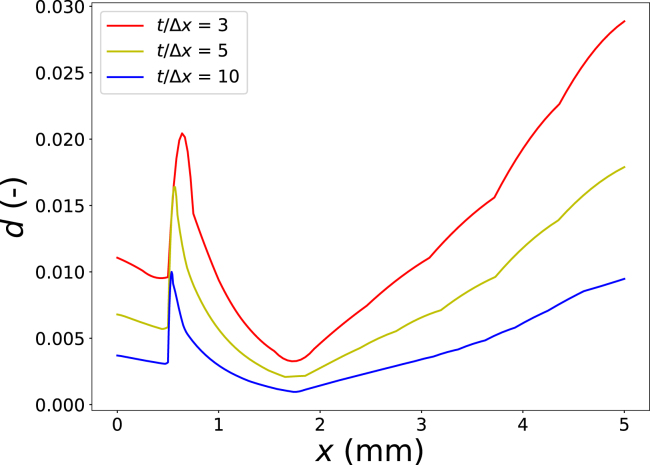
Comparison of the damage field along the central cross section (y=0) for different grid resolutions, expressed as the ratio t/Δx.

We performed such a study by varying the grid resolution through the ratio t/Δx, where t denotes the crust thickness, while keeping the ratio Δx/L=4 constant. The damage field was evaluated along the horizontal line intersecting the centre of the square cross section (i.e. y=0) for t/Δx={3,5,10}. The results are shown in Fig. 8. With increasing grid resolution, the gradients in the damage field become sharper; however, a fully converged solution is not yet achieved. Achieving convergence would require substantially finer meshes and correspondingly longer computation times.

The aim of the present study is not to obtain quantitatively precise predictions, but rather to estimate the likelihood and spatial localization of freezing-induced damage. The observed trends with increasing grid resolution nevertheless confirm the occurrence of microdamage, particularly at the crust–core interface, in agreement with our expectations.

**Fig. 9:**
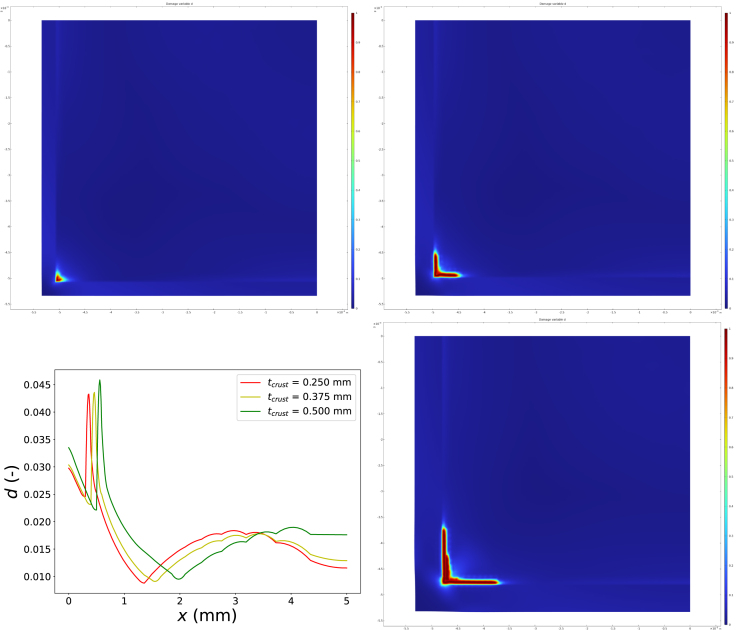
Damage field d for crust thicknesses $t_{\mathrm{crust}}=\left\{0.25,0.375,0.50\right\}\;\mathrm{mm}$ (from top left to bottom right), and the corresponding damage profiles along the central cross section (y=0) (bottom left).

In a final series of simulations, we varied key physical parameters. First, we varied the crust thickness, which in practice reflects changes in par-frying time or temperature (van der Sman and van den Oudenhoven, 2023). For these simulations, we set $T_{\mathrm{air}}=-13{\;}^{\circ }C$, $\epsilon_{w0,\mathrm{crust}}=0.56$, and $t_{e}=550$ s. The results are shown in Fig. 9. We observe crack formation, which becomes more pronounced as the crust thickness increases. This trend is consistent with our experimental observations, which showed that freezing damage increases with more extensive par-frying (van der Sman and van den Oudenhoven, 2023).

In these simulations, damage localized at the corner between the crust and core develops into a macroscopic crack, with d=1, which subsequently propagates along the crust–core interface. In practice, the par-fried crust does not fracture off during freezing; however, these results are indicative of the high sensitivity of the par-fried system to fracture. The propensity for cracking also depends strongly on the fracture energy of the ice inclusions, $G_{c,\mathrm{ice}}$, which was set to $G_{c,\mathrm{ice}}=10$ J m^−2^ in this crust-thickness sweep. As noted earlier, $G_{c,\mathrm{ice}}$ is one of the most uncertain parameters governing fracture in frozen fries.

**Fig. 10:**
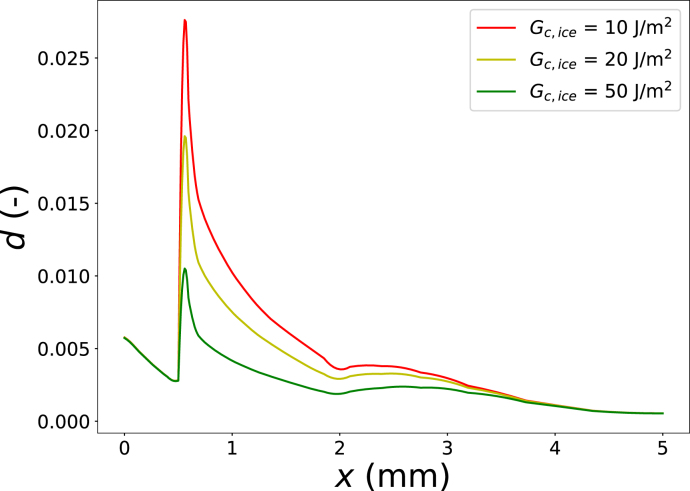
Comparison of the damage field along the central cross section (y=0) for different fracture energies of ice: $G_{c,ice}=\left\{10,20,50\;{\mathrm{J/m}}^{2}\right\}$.

We therefore performed additional simulations in which $G_{c,\mathrm{ice}}$ was varied over the range $10\leq G_{c,\mathrm{ice}}\leq 50$ J m^−2^, while keeping $T_{\mathrm{air}}=-13{\;}^{\circ }C$, $\epsilon_{w0,\mathrm{crust}}=0.56$, and $t_{e}=550$ s. The resulting damage fields along the central cross section are shown in Fig. 10. Freezing-induced damage decreases markedly with increasing $G_{c,\mathrm{ice}}$, and macroscopic fracture is absent for $G_{c,\mathrm{ice}}\geq 20$ J m^−2^.

**Fig. 11:**
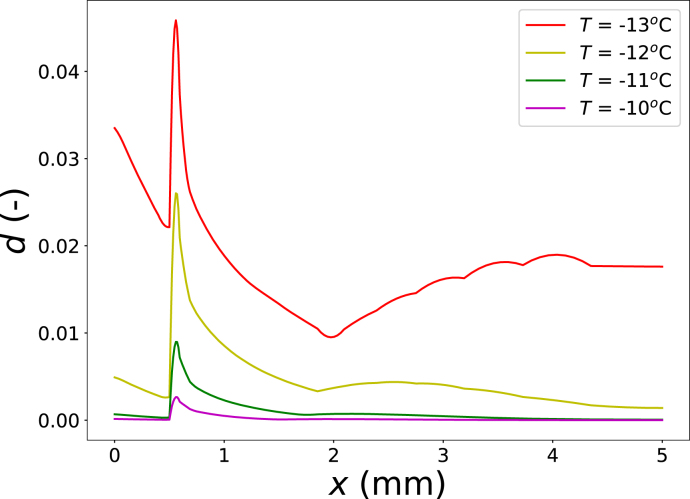
Comparison of the damage field along the central cross section (y=0) for different freezing temperatures.

Next, we varied the freezing temperature, $T_{\mathrm{air}}=\left\{-13,-12,-11,-10{\;}^{\circ }C\right\}$, while keeping $\epsilon_{w0,\mathrm{crust}}=0.56$ and $G_{c,\mathrm{ice}}=20$ J m^−2^. The damage fields along the central cross section at the end of freezing ($t_{e}=550$ s) are shown in Fig. 11. Lower freezing temperatures lead to increased damage, reflecting enhanced ice formation and correspondingly higher thermal stresses. This trend is again consistent with experimental observations, where freezing damage was found to increase with decreasing freezing temperature (van der Sman and van den Oudenhoven, 2023).

## 4 Conclusions

In this study, we evaluated the fracture phase field method as a modelling framework for investigating freezing damage, a phenomenon that remains poorly understood in food and plant science. As a case study, we considered damage occurring during the freezing of par-fried French fries, for which experimental observations were reported in an earlier study.

Fracture properties of food materials are rarely available in the literature. We therefore assumed that the starch-rich fry material behaves similarly to cereal flakes. For this latter material, we demonstrated that the fracture energy scales with $T_{g}/T$, consistent with earlier findings for other rheological properties of starch-based materials. Frozen fries were treated as a composite material consisting of a (de)hydrated starch matrix containing ice crystals. The fracture energy of ice crystals embedded in a frozen food matrix is largely unknown; in this study, it was assumed to lie in the range $10<G_{c,\mathrm{ice}}<50$ J m^−2^.

Our simulations indicate that microdamage is likely to develop at the interface between the partially unfrozen crust and the frozen core. For low values of $G_{c,\mathrm{ice}}=10$ J m^−2^, the simulations predicted the formation of macroscopic cracks at the crust–core interface, which were not observed experimentally. Such cracks were absent for $G_{c,\mathrm{ice}}\geq 20$ J m^−2^. Within this parameter range, freezing damage decreased with increasing freezing temperature and decreasing crust thickness, in agreement with trends observed in our experimental study.

This work is intended as a first evaluation of the fracture phase field method for frozen food materials. Based on the present results, we regard it as a promising modelling approach, as the simulations reproduce experimentally observed trends in freezing damage. It should be noted, however, that the initial conditions of the par-fried fries were idealized by imposing a sharp discontinuity in moisture content between crust and core. In reality, this transition is more gradual and would require explicit simulation of the par-frying process itself. Such modelling necessitates a proper description of the dynamically changing heat transfer associated with steam bubble formation, which remains an open research challenge (Costa et al., 1999, Van Koerten et al., 2017). Addressing this issue will be the subject of future work. In addition, the frozen material was assumed to behave as purely elastic, whereas experimental results indicate the presence of viscoelastic relaxation (van der Sman and van den Oudenhoven, 2023). In the deeply frozen state, such relaxation processes are slow, with characteristic timescales on the order of weeks. In the unfrozen or partially frozen state, however, relaxation is expected to be significantly faster, as it also scales with $T_{g}/T$ (Van der Sman et al., 2022). Consequently, viscoelasticity is expected to influence fracture behaviour (Dammaß et al., 2023) and should be incorporated in future extensions of the model.

Finally, for a more comprehensive understanding of freezing damage, it is important to consider the phenomenon of cryosuction (Coussy, 2005). Recent studies have shown that cryosuction plays a crucial role in fracture formation in frozen hydrogels (Yang et al., 2024), suggesting that damage may arise not only from thermal stresses, but also from dehydration of the matrix ahead of advancing ice crystals. Since food materials can often be idealized as hydrogel-like systems (Van der Sman et al., 2013, Jin and van der Sman, 2022), this mechanism may also be highly relevant for freezing damage in foods. The phase field framework is particularly well suited for addressing this problem, as it can capture both ice crystal formation (van der Sman, 2021) and fracture processes (Dammaß et al., 2023) within a unified modelling approach.

## Declaration of competing interest

I declare no conflicts of interest, except I have been recently a guest editor of special issue of CRFS.
